# Geographic variation and sociodemographic correlates of prescription psychotropic drug use among children and youth in Ontario, Canada: a population-based study

**DOI:** 10.1186/s12889-022-14677-6

**Published:** 2023-01-11

**Authors:** Tony Antoniou, Daniel McCormack, Sophie Kitchen, Kathleen Pajer, William Gardner, Yona Lunsky, Melanie Penner, Mina Tadrous, Muhammad Mamdani, David N. Juurlink, Tara Gomes

**Affiliations:** 1 Li Ka Shing Knowledge Institute, St. Michael’s Hospital, Toronto, Ontario Canada; 2 ICES, Toronto, Ontario Canada; 3 Department of Family and Community Medicine, University of Toronto, Toronto, Ontario Canada; 4 Department of Family and Community Medicine, St. Michael’s Hospital, Toronto, Ontario Canada; 5 Children’s Hospital of Eastern Ontario Research Institute, Ottawa, Ontario Canada; 6 Department of Psychiatry, University of Ottawa, Ottawa, Ontario Canada; 7 School of Epidemiology and Public Health, University of Ottawa, Ottawa, Ontario Canada; 8 Azrieli Adult Neurodevelopmental Centre, Centre for Addiction and Mental Health, Toronto, Canada; 9 Department of Psychiatry, University of Toronto, Toronto, Ontario Canada; 10 Autism Research Centre, Bloorview Research Institute, Holland Bloorview Kids Rehabilitation Hospital, Toronto, Canada; 11 Department of Pediatrics, University of Toronto, Toronto, Ontario Canada; 12 Leslie Dan Faculty of Pharmacy, University of Toronto, Toronto, Ontario Canada; 13 Li Ka Shing Centre for Healthcare Analytics Research & Training, Unity Health Toronto, Toronto, Ontario Canada; 14 Temerty Faculty of Medicine, University of Toronto, Toronto, Ontario Canada; 15 Institute of Health Policy, Management, and Evaluation, University of Toronto, Toronto, Ontario Canada; 16 Department of Medicine, University of Toronto, Toronto, Ontario Canada

**Keywords:** Child, Adolescent, Psychotropic drugs, Prescriptions / statistics & numerical data, Geographic variation, Small-area analysis, Socioeconomic factors

## Abstract

**Background:**

Population-based research examining geographic variability in psychotropic medication dispensing to children and youth and the sociodemographic correlates of such variation is lacking. Variation in psychotropic use could reflect disparities in access to non-pharmacologic interventions and identify potentially concerning use patterns.

**Methods:**

We conducted a population-based study of all Ontario residents aged 0 to 24 years who were dispensed a benzodiazepine, stimulant, antipsychotic or antidepressant between January 1, 2018, and December 31, 2018. We conducted small-area variation analyses and identified determinants of dispensing using negative binomial generalized estimating equation models.

**Results:**

The age- and sex-standardized rate of psychotropic dispensing to children and youth was 76.8 (range 41.7 to 144.4) prescriptions per 1000 population, with large variation in psychotropic dispensing across Ontario’s census divisions. Males had higher antipsychotic [rate ratio (RR) 1.40; 95% confidence interval (CI) 1.36 to 1.44) and stimulant (RR 1.75; 95% CI 1.70 to 1.80) dispensing rates relative to females, with less use of benzodiazepines (RR 0.85; 95% CI 0.83 to 0.88) and antidepressants (RR 0.81; 95% CI 0.80 to 0.82). Lower antipsychotic dispensing was observed in the highest income neighbourhoods (RR 0.72; 95% CI 0.70 to 0.75) relative to the lowest. Benzodiazepine (RR 1.12; 95% CI 1.01 to 1.24) and stimulant (RR 1.11; 95% CI 1.01 to 1.23) dispensing increased with the density of mental health services in census divisions, whereas antipsychotic use decreased (RR 0.82; 95% CI 0.73 to 0.91). The regional density of child and adolescent psychiatrists and developmental pediatricians (RR 1.00; 95% CI 0.99 to 1.01) was not associated with psychotropic dispensing.

**Conclusion:**

We found significant variation in psychotropic dispensing among young Ontarians. Targeted investment in regions with long wait times for publicly-funded non-pharmacological interventions and novel collaborative service models may minimize variability and promote best practices in using psychotropics among children and youth.

**Supplementary Information:**

The online version contains supplementary material available at 10.1186/s12889-022-14677-6.

## Introduction

The use of psychotropic medication, such as stimulants, antipsychotics, benzodiazepines and antidepressants, has increased among Canadian children and youth, with at least 1 in 15 receiving a psychotropic drug between 2012 and 2017 [1, 2]. Similar trends have been observed in Europe and the United States [3–5]. Yet, little is known about geographic variability in the use of these drugs among children and youth and the sociodemographic correlates of such variation. While such variability may reflect regional differences in the rates of mental health conditions, geographic variation in prescription psychotropic drug use among children and youth could also reflect regional disparities in access to evidence-based non-pharmacological therapies, uneven distribution of mental health care professionals, particularly in rural and remote settings, and inappropriate overuse or underuse of these drugs in specific treatment settings [6–10]. Specifically, several studies in the United States have documented low receipt of psychosocial treatment among Medicaid-insured children and youth treated with psychotropic drugs, with 49% of individuals less than 20 years of age receiving such treatment prior to initiating antipsychotics and less than 38% of children with attention deficit/hyperactivity disorder (ADHD) receiving any psychotherapy prior to initiating treatment. In the absence of psychosocial services, medications may become the default intervention [11, 12]. Given that access to such services may be limited by geographic accessibility and cost [13], and that pharmacological therapies incur the risk of serious adverse effects, studies exploring geographic variability in psychotropic drug use among children and youth and the correlates of such use are needed to inform programming and policy for children and youth with mental health conditions.

However, available research has focused mainly on variation in stimulant treatment across the United States or among specific populations of children and youth defined by age category and child welfare system contact, with few comparisons of the different psychotropic drug classes at a population-level [14–22]. Moreover, some studies have only been able to examine psychotropic use among children and youth with specific forms of drug insurance, and therefore selectively exclude large groups of children and youth receiving treatment [17, 23–25]. Hence, inferences from these studies may not be generalizable to children and youth not represented in these databases, and thereby limit opportunities to understand local patterns and variation in psychotropic drug use in specific regions. Studies encompassing the entire population of children and youth are needed to explore variation in psychotropic drug use and identify opportunities for policy and educational intervention.

Between January 2018 and March 2019, all children and youth aged 24 and under in Ontario, Canada were eligible to receive prescription medication at no cost through a publicly-funded universal pharmacare program [26]. Prescription drug data were therefore available for the entire population of individuals aged 24 and under in Ontario during this period. This represented a unique opportunity to study variation in prescription psychotropic drug use among children and youth that was not confounded by drug insurance status. Accordingly, we studied geographic variability in prescription psychotropic drug use among the entire population of individuals aged 0 to 24 in Ontario, home to approximately 4.1 million children and youth [27]. Our objectives were to quantify the extent of geographic variability in prescription psychotropic dispensing to children and youth and identify sociodemographic correlates of use.

## Methods

### Setting

We conducted a population-based study of all Ontario residents between 0 and 24 years of age who were dispensed a benzodiazepine, stimulant, antipsychotic, or antidepressant drug between January 1, 2018 and December 31, 2018. These individuals had universal access to prescription drug coverage, hospital care, and physicians’ services. Our study was conducted at the level of Ontario census divisions, which represent a group of neighbouring municipalities designated as counties or regional districts for the purpose of regional planning and distribution of services. There were 49 census divisions in Ontario during the study period, ranging in total population from approximately 13,000 to 2,700,000 (3500 to 738,000 individuals aged 0 to 24) in 2016 [28].

### Data sources

We identified claims for psychotropics using the Ontario Drug Benefit (ODB) database, which contains comprehensive records of all publicly-funded medications dispensed to Ontario residents. To ascertain patient comorbidity and the prevalence of antipsychotic-treated children and youth with diagnoses of schizophrenia and other psychotic disorders or autism spectrum disorder, we used the Ontario Health Insurance Plan (OHIP) database to identify claims for physician services. We also obtained diagnostic information from inpatient hospital admissions, emergency department visits, and mental health-related hospitalizations using the Canadian Institute for Health Information’s Discharge Abstract Database, National Ambulatory Care Reporting System database, and Ontario Mental Health Reporting System database, respectively.

To determine if variation in psychotropic use was associated with the regional density of medical mental health and neurodevelopmental specialists, we used the ICES Physician Database to identify all active child and adolescent psychiatrists and developmental pediatricians in Ontario. We considered both specialists in a composite of medical mental health expertise because mental health conditions such as mood and psychotic disorders are typically treated by child and adolescent psychiatrists, whereas children and youth with neurodevelopmental conditions are typically treated by developmental pediatricians. We used the Registered Persons Database, a registry for all individuals eligible for Ontario health insurance, to determine demographic characteristics. These datasets were linked using unique encoded identifiers and analyzed at ICES in Toronto, Ontario (https://www.ices.on.ca). The use of data in this project is authorized under section 45 of Ontario’s Personal Health Information Protection Act, which does not require review by a Research Ethics Board.

### Statistical analysis

We calculated psychotropic dispensing rates as the number of prescriptions dispensed for benzodiazepines, stimulants, antidepressants and antipsychotics per 1000 Ontarians aged 0 to 24 years in each census division. Next, we conducted several analyses to measure variation in psychotropic dispensing rates across census divisions [29]. First, we determined the extremal quotient (EQ), defined as the ratio of the highest psychotropic dispensing rate to the lowest among the census divisions. Second, we determined the coefficient of variation (CV), defined as the ratio of the standard deviation of census division dispensing rates to the mean census division dispensing rate, weighted by the population in each census division to account for their unequal population sizes. We also determined the systematic component of variation (SCV), which measures the relative systematic component of variation in rates among census divisions by subtracting the random component of variance from the total variance. The SCV is therefore an estimate of the ‘true’ non-random variation, with values of 3.0 to 5.4, 5.5 to 10 and greater than 10 indicating moderate, high, and very high variation, respectively [29, 30]. Finally, we compared individual census division dispensing rates with the rate for the province as a whole using chi-square tests, adjusting for multiple comparisons using a Type 1 error threshold of 0.001 (approximately 0.05/49) to adjust for multiple comparisons.

To identify sociodemographic correlates of variation in use, we used generalized estimating equations with a log-link function and exchangeable correlation structure to account for correlation within PHUs [31]. We conducted these analyses using the entire population of children and youth between the ages of 0 and 24 who had received a psychotropic drug during the study period and a 10% random sample of the remaining population of Ontario residents aged 24 and under. We used a 10% random sample of children and youth who were not prescribed a psychotropic drug to minimize the computational burden associated with including the entire population of these children and youth. The outcome variable was the number of individuals dispensed psychotropic drugs in each census division over the study period, using the census division population size of children and youth aged 24 and under as an offset term. We defined individual-level characteristics on the first dispensing date for recipients of psychotropic drugs and a randomly assigned index date during the study period for non-recipients. Characteristics considered for inclusion in models were those that we theorized might influence psychotropic need and access based on prior research, including age, sex, rural versus urban residence, and socioeconomic status, estimated at the neighbourhood level using postal code information and Statistics Canada census data.

We ascertained individual comorbidity in the prior 2 years using the Johns Hopkins ACG® System Version 10 case-mix assignment software. We specifically used ACG® System Aggregated Diagnosis Groups (ADGs), which are clusters of diagnostic codes with similar severity and expected persistence [32]. The count of ADGs ranges from 0 to 32, with higher values reflecting more comorbidity. Although we could not derive ADGs for individuals less than 2 years of age with this approach, these children comprised only 0.01% of the cohort. We also included census division characteristics that we theorized could influence access to or need for psychotropic medication, including the number of child and adolescent psychiatrists and developmental pediatricians, the number of facilities providing mental health care to children and youth (including hospital-based inpatient and outpatient programs and community children’s mental health agencies), and the regional rate of mental health-related hospitalizations and emergency department visits among children and youth. Finally, we used information from the 2016 census to derive six social and economic characteristics of census divisions, including the mean household size and the proportions of the population with post-secondary education, identifying as a visible minority, non-Canadian citizens, employed, and those who speak neither English nor French. We expressed the impact of individual- and census division-level variables as rate ratios (RR) and their 95% confidence intervals (CIs), defined as the prescribing rate in a category (e.g., rural) relative to the rate in the referent category (e.g., urban). We stratified analyses by psychotropic drug class to explore heterogeneity in determinants of prescribing among individuals undergoing treatment with antipsychotics, stimulants, benzodiazepines, and antidepressants, using generalized estimating equations with a log-link function, independent correlation structure and robust standard errors with clustering at the census division level. All analyses were conducted using SAS Enterprise Guide version 7.1 (SAS Institute, Cary, North Carolina, USA).

## Results

We identified 306,470 children and youth who received psychotropic medication between January 1, 2018 and December 31, 2018. Compared with a random 10% sample of the general population not dispensed a psychotropic during this period (*N* = 371,800), psychotropic medication recipients were older (median age 18 years; interquartile range [IQR] 14 to 21 years versus 12 years; IQR 6 to 19 years; standardized difference (SD) = 0.75) and had a greater comorbidity burden as reflected by the proportion of individuals with ten or more ADGs (15.3% vs. 4.1%; SD = 0.39) (Table 1).

**Table 1:** Baseline characteristics

Variable	Psychotropic recipients (***n*** = 306,470)	Non-psychotropic recipients^**a**^ (***n*** = 371,800)	Standardized Difference
Age (median, IQR)	18 (14–21)	12 (6–19)	0.75
0–4	1175 (0.4%)	66,067 (17.8%)	0.63
5–9	30,060 (9.8%)	76,377 (20.5%)	0.30
10–14	50,826 (16.6%)	76,773 (20.6%)	0.10
15–19	98,517 (32.1%)	72,626 (19.5%)	0.29
20–24	125,892 (41.1%)	79,957 (21.5%)	0.43
Female, No. (%)	159,740 (52.1%)	179,670 (48.3%)	0.08
Income quintile			
1 (lowest)	62,279 (20.3%)	73,710 (19.8%)	0.01
2	57,947 (18.9%)	68,804 (18.5%)	0.01
3	57,269 (18.7%)	73,995 (19.9%)	0.03
4	60,796 (19.8%)	77,411 (20.8%)	0.02
5	68,179 (22.2%)	77,880 (20.9%)	0.03
Residence			
Urban	271,467 (88.6%)	336,659 (90.5%)	0.06
Rural	35,003 (11.4%)	35,141 (9.5%)	0.06
ADG Category			
0–5	147,812 (48.2%)	272,240 (73.2%)	0.53
6–9	111,880 (36.5%)	84,390 (22.7%)	0.31
> 10	46,778 (15.3%)	15,170 (4.1%)	0.39

^a^10% random sample of children and youth who were not dispensed a psychotropic medication

When stratified by psychotropic class, the majority of benzodiazepine and antidepressant recipients were female (63.2 and 64.9%, respectively), while antipsychotic and stimulant recipients were predominantly male (53.7 and 68.8%, respectively) (Supplemental Table 1). Furthermore, a socioeconomic gradient was observed in the use of stimulants, with greater use in the highest relative to the lowest income neighbourhoods (24.4% vs. 18.9%), with the converse being true for antipsychotics (17.3% vs. 26.0%) (Supplemental Table 1). As expected, children less than 5 years of age comprised the smallest proportion of children and youth receiving psychotropic drugs. In addition, benzodiazepine and antipsychotic recipients had a greater comorbidity burden than individuals receiving other classes, with nearly 1 in 4 individuals in both groups having 10 or more aggregated diagnosis groups (Supplemental Table 1).

### Regional variation

**Fig. 1:**
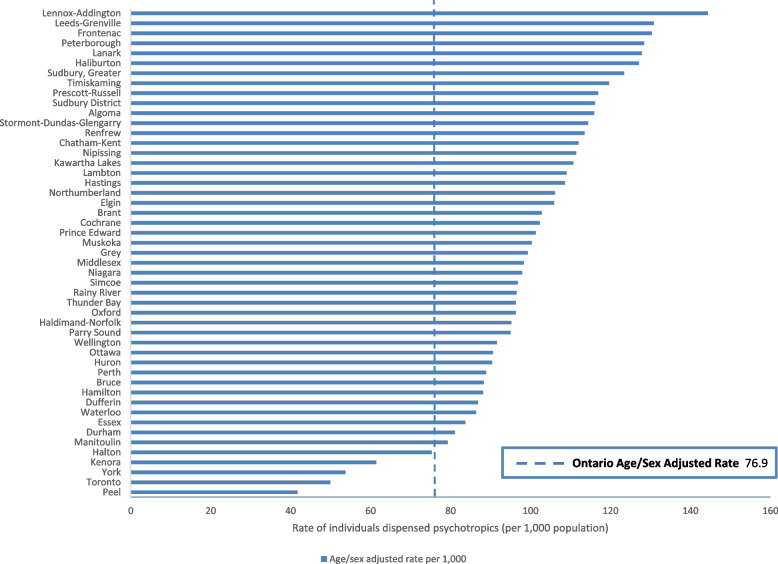
Age- and sex-adjusted rates of individuals dispensed psychotropics by census division (per 1000 population)

The overall crude and age- and sex-adjusted rates of psychotropic dispensing in Ontario were 74.9 (range 40.1 to 139.8) and 76.9 (range 41.7 to 144.4) per 1000 population, respectively (Fig. 1). Five predominantly urban census divisions representing 44.2% of the provincial population of children and youth had psychotropic dispensing rates that were significantly lower than the provincial average (*p* < 0.001), while 43 census divisions representing 55.7% of the provincial population had rates significantly higher than the provincial average (*p* < 0.001).

The age- and sex-adjusted rates for antidepressants, stimulants, antipsychotics and benzodiazepines were 47.8 per 1000, 28.4 per 1000, 13.5 per 1000 and 9.7 per 1000, respectively (Supplemental Figs. 1, 2, 3 and 4). There was a moderate correlation between psychotropic dispensing rates and the density of clinics and agencies providing mental health care for children and youth (Spearman’s rho 0.52; 95% CI 0.27 to 0.71) and rates of mental health hospitalizations and emergency department visits (Spearman’s rho 0.48; 95% CI 0.22 to 0.68). Conversely, there was a weak negative correlation between psychotropic dispensing rates and the density of child and adolescent psychiatrists and developmental pediatricians (Spearman’s rho − 0.32; 95% CI − 0.56 to − 0.03). The EQ, CV, and SCV were 3.5, 33.2 and 167.5, respectively, indicating large variations in the rates of psychotropic prescribing in children and youth across Ontario’s census divisions following adjustment for the age and sex composition of the population. Results were similar when stratified by psychotropic class (Supplemental Table 2).

### Correlates of variation

Following multivariable adjustment, individual-level variables most strongly associated with receiving a psychotropic included age and comorbidity burden (Table 2). Specifically, compared with individuals between 5 and 9 years of age, rates of psychotropic prescribing were lower among individuals between the ages of 0 to 4 years (RR 0.05; 95% CI 0.04 to 0.06), and higher among those aged 10 to 14 years (RR 1.43; 95% CI 1.37 to 1.49), 15 to 19 years (RR 1.83; 95% CI 1.71 to 1.96) and 20 to 24 years (RR 1.88; 95% CI 1.73 to 2.03). Similar findings were observed when analyses were stratified by psychotropic class, with the exception of stimulants, where rates were highest in individuals aged 10 to 14 years (RR 1.11; 95% CI 1.08 to 1.14) relative to individuals between the ages of 5 and 9. In addition, psychotropic dispensing rates were higher among individuals with high comorbidity burden. Specifically, those with 6 to 9 aggregated diagnosis groups (RR 1.58; 95% CI 1.54 to 1.63) and 10 or more (RR 1.88; 95% CI 1.78 to 1.97) aggregated diagnosis groups had higher dispensing rates relative to individuals with five or fewer aggregated diagnosis groups.

**Table 2:** Individual and County Variables Associated with Psychotropic Dispensing to Children and Youth in Ontario, January 1, 2018, to December 31, 2018

Variable	All psychotropics (Adjusted rate ratio, 95% CI)	Antidepressants (Adjusted rate ratio, 95% CI)	Antipsychotics (Adjusted rate ratio, 95% CI)	Benzodiazepines (Adjusted rate ratio, 95% CI)	Stimulants (Adjusted rate ratio, 95% CI)
**Individual-level variables**					
Age					
0–4	0.05 (0.04 to 0.06)	0.02 (0.02 to 0.03)	0.14 (0.11 to 0.19)	0.46 (0.36 to 0,59)	0.04 (0.03 to 0.05)
5–9 (ref)	1.00	1.00	1.00	1.00	1.00
10–14	1.43 (1.37 to 1.49)	4.56 (4.09 to 5.08)	1.25 (1.18 to 1.32)	2.89 (2.55 to 3.27)	1.11 (1.08 to 1.14)
15–19	1.83 (1.71 to 1.96)	8.73 (7.60 to 10.03)	1.53 (1.38 to 1.69)	8.60 (7.39 to 10.01)	0.81 (0.77 to 0.85)
20–24	1.88 (1.73 to 2.03)	9.32 (8.05 to 10.79)	1.80 (1.58 to 2.05)	12.37 (10.50 to 14.58)	0.61 (0.55 to 0.67)
Sex					
Male	1.13 (1.11 to 1.15)	0.81 (0.80 to 0.82)	1.40 (1.36 to 1.44)	0.85 (0.83 to 0.88)	1.75 (1.70 to 1.80)
Female (ref)	1.00	1.00	1.00	1.00	1.00
Rurality					
Rural	0.93 (0.90 to 0.96)	0.95 (0.92 to 0.98)	0.90 (0.86 to 0.94)	0.94 (0.91 to 0.98)	0.91 (0.87 to 0.95)
Urban (ref)	1.00	1.00	1.00	1.00	1.00
Neighbourhood income quintile					
1 (ref)	1.00	1.00	1.00	1.00	1.00
2	0.97 (0.95 to 0.99)	1.01 (0.99 to 1.02)	0.88 (0.86 to 0.91)	1.01 (0.99 to 1.04)	0.97 (0.93 to 1.01)
3	0.93 (0.90 to 0.96)	0.98 (0.96 to 1.00)	0.81 (0.78 to 0.84)	0.98 (0.95 to 1.02)	0.95 (0.91 to 1.00)
4	0.92 (0.89 to 0.96)	0.98 (0.95 to 1.01)	0.75 (0.72 to 0.79)	1.00 (0.97 to 1.03)	0.95 (0.90 to 1.01)
5	0.92 (0.88 to 0.97)	0.97 (0.93 to 1.00)	0.72 (0.70 to 0.75)	1.01 (0.98 to 1.04)	1.00 (0.92 to 1.09)
ADG category					
0–5	1.00	1.00	1.00	1.00	1.00
6–9	1.58 (1.54 to 1.63)	1.53 (1.51 to 1.55)	1.77 (1.71 to 1.82)	1.82 (1.76 to 1.88)	1.5 (1.47 to 1.53)
> 10	1.88 (1.78 to 1.97)	1.73 (1.68 to 1.77)	2.33 (2.18 to 2.49)	2.61 (2.47 to 2.76)	1.71 (1.64 to 1.78)
Other psychotropic class		2.16 (2.03 to 2.3)	10.33 (9.64 to 11.07)	6.66 (6.16 to 7.2)	2.65 (2.51 to 2.79)
**County-Level Variables**					
Pediatric psychiatry/developmental pediatrician (count per 100,000)	1.00 (0.99 to 1.01)	1.00 (1.00 to 1.01)	1.00 (0.99 to 1.01)	1.00 (0.99 to 1.01)	1.01 (1.00 to 1.02)
Children and youth mental health agencies/services (per 1000 population)	1.07 (1.00 to 1.13)	0.98 (0.91 to 1.05)	0.82 (0.73 to 0.91)	1.12 (1.01 to 1.24)	1.11 (1.01 to 1.23)
Children and youth mental health hospital admissions/emergency department visits (per 1000 population)	1.00 (0.99 to 1.00)	1.00 (1.00 to 1.00)	1.00 (1.00 to 1.01)	0.99 (0.99 to 1.00)	0.99 (0.99 to 1.00)
% population with post-secondary education	1.01 (1.00 to 1.01)	1.00 (1.00 to 1.01)	1.00 (0.99 to 1.00)	1.00 (0.99 to 1.00)	1.01 (1.01 to 1.02)
% population that recognizes as visible minority	0.99 (0.99 to 0.99)	1.00 (0.99 to 1.00)	1.00 (0.99 to 1.00)	0.99 (0.98 to 1.00)	1.00 (0.99 to 1.00)
Mean number of persons per private household	0.90 (0.78 to 1.04)	0.83 (0.71 to 0.97)	0.88 (0.7 to 1.11)	1.34 (1.04 to 1.71)	0.74 (0.62 to 0.87)
% population that are not Canadian citizens	1.01 (0.99 to 1.02)	0.99 (0.97 to 1.01)	1.00 (0.98 to 1.02)	1.03 (1.00 to 1.07)	0.96 (0.94 to 0.97)
% population that is employed	1.00 (1.00 to 1.01)	1.00 (1.00 to 1.01)	1.00 (0.99 to 1.00)	0.99 (0.99 to 1.00)	1.00 (1.00 to 1.01)
% population that speaks neither English nor French	1.00 (0.98 to 1.01)	0.98 (0.96 to 0.99)	0.95 (0.93 to 0.97)	0.96 (0.94 to 0.99)	1.03 (1.01 to 1.04)

Although the use of psychotropic drugs was higher in males than females (RR 1.13; 95% CI 1.11 to 1.15), this finding was driven chiefly by higher rates of antipsychotic (RR 1.40; 95% CI 1.36 to 1.44) and stimulant (RR 1.75; 95% CI 1.70 to 1.80) use, with lower rates of benzodiazepine (RR 0.85; 95% CI 0.83 to 0.88) and antidepressant (RR 0.81; 95% CI 0.80 to 0.82) dispensing relative to females. A socioeconomic gradient was also observed, in which psychotropic dispensing rates were lower in the highest income neighbourhood (RR 0.92; 95% CI 0.88 to 0.97) relative to the lowest. This finding was driven mostly by variation in antipsychotics, the dispensing of which was 28% lower in the highest relative to the lowest income neighbourhoods (17.3% vs. 26.0%; RR 0.72, 95% 0.70 to 0.75). The proportion of antipsychotic-treated children and youth with a diagnosis of schizophrenia or other psychotic disorder in the 30- and 365-days prior to being dispensed a prescription for an antipsychotic was 16.6% (*n* = 8919) and 18,387 (34.2%), respectively, with little variation according to neighbourhood income quintile (Supplemental Table 3). Specifically, the proportions of antipsychotic-treated children and youth in the lowest and highest income neighbourhoods with a diagnosis of schizophrenia or other psychotic disorder in the 30 days prior to the antipsychotic dispensing date were 17.3% (2414/13,963) and 16.1% (1503/9329), respectively. Respective figures for a diagnosis of schizophrenia or other antipsychotic disorder within 365 days of the dispensing date were 34.5% (4851/13,963) and 33.7% (3141/9329) (Supplemental Table 3). The proportions of antipsychotic-treated children and youth with a diagnosis of autism spectrum disorder in the 30- and 365-days preceding the antipsychotic dispensing date were 3.2% (*n* = 1731) and 9.7% (*n* = 5205), respectively (Supplemental Table 3). In analyses stratified by neighbourhood income quintile, the proportions of antipsychotic-treated children and youth in the lowest and highest income neighbourhoods with a diagnosis of autism spectrum disorder in the 30 days prior to the antipsychotic dispensing date were 3.0% (421/13,963) and 3.3% (307/9329), respectively. Respective figures for a diagnosis of autism spectrum disorder within 365 days of the dispensing date were 8.9% (1241/13,963) and 10.3% (964/9329) (Supplemental Table 3).

Census division characteristics were also associated with psychotropic prescribing. Specifically, benzodiazepine (RR 1.12; 95% CI 1.01 to 1.24) and stimulant (RR 1.11; 95% CI 1.01 to 1.23) dispensing increased with the density of mental health services in census divisions, whereas antipsychotic use decreased (RR 0.82; 95% CI 0.73 to 0.91). Stimulant dispensing was also inversely associated with the mean number of individuals per household (RR 0.74; 95% CI 0.62 to 0.87) and the proportion of the census division population that are not Canadian citizens (RR 0.96; 95% CI 0.94 to 0.97). Conversely, benzodiazepine dispensing increased with household size (RR 1.34; 95% CI 1.04 to 1.71) and the proportion of the population that are not Canadian citizens (RR 1.03; 95% CI 1.00 to 1.07). Census division characteristics that were not associated with psychotropic dispensing included the regional density of child and adolescent psychiatrists and developmental pediatricians (RR 1.00; 95% CI 0.99 to 1.01) and regional rates of mental health hospital admissions and emergency department visits per 1000 children and youth (RR 1.00; 95% CI 0.99 to 1.00) (Table 2).

## Discussion

In our population-based study, we found substantial variation in the magnitude and nature of psychotropic dispensing among Ontario children and youth, with demographic factors, comorbidity burden, socioeconomic status, and the availability of mental health services being important determinants of use. Although we observed a positive correlation between psychotropic dispensing and regional rates of mental health-related hospitalizations and emergency department visits, this association was not apparent following multivariable adjustment. Similarly, the regional density of child and adolescent psychiatrists and developmental pediatricians was not associated with psychotropic dispensing. Overall, our findings suggest that important disparities may exist in the use of psychotropics among children and youth despite universal access to medications and medical mental health services.

Our findings add to earlier studies exploring variation in prescription psychotropic drug use among children and youth. Although our findings associating psychotropic prescribing rates with age, male sex and comorbidity are similar to those of prior studies [33–36], we additionally quantified the extent and determinants of variation by individual drug classes, thereby allowing us to identify medication use patterns that are otherwise obscured when examining only psychotropic dispensing as a whole. Similarly, most prior studies have focused on geographic variability in stimulant use across the United States or variation in psychotropic dispensing across Canadian provinces [14–17, 37]. In contrast, we explored small-area variation in the use of four classes of psychotropic drugs in a single Canadian province. In addition, previous studies have focused on specific subpopulations of children and youth that vary according to certain characteristics, such as health insurance status and contact with the child welfare system. In contrast, we studied the entire population of children and youth treated with psychotropic drugs. Because these individuals had access to publicly financed health care, including prescription drugs, our work extends the study of geographic variability in psychotropic use among children and youth to a setting with universal health insurance. Finally, although other studies have examined population-wide psychotropic drug use in children and youth, they have generally been focused on evaluating trends over time [5, 38–40]. Conversely, we focused on geographic variation identify patterns of psychotropic use and its correlates in the largest population of children and youth in Canada.

Several mechanisms may explain the patterns of psychotropic dispensing observed in our study. Specifically, antipsychotic prescribing rates were inversely associated with neighbourhood income, and were highest in the lowest income neighbourhoods of Ontario. This is important, because antipsychotics are primarily used for the management of non-psychotic disorders and externalizing symptoms in children and youth [41, 42], require regular monitoring for adverse metabolic effects [43], and have been associated with an increased risk of death [44]. Our finding correlating psychotropic dispensing with mental health related hospitalizations and emergency department visits suggests that some variation in dispensing could reflect need and variation in rates of mental health conditions according to region and/or socioeconomic status. However, it does not appear that variation in need alone explains the variability in antipsychotic dispensing by socioeconomic status, as the proportion of antipsychotic-treated children and youth with a diagnosis of schizophrenia or other psychotic disorder in the 30 and 365 days preceding their prescription was similar across neighbourhood income quintile groups. In contrast, the prevalence of stimulant prescribing was greatest among children living in the highest-income neighbourhoods. This finding is consistent with past research demonstrating lower treatment rates in low-income children with attention-deficit hyperactivity disorder (ADHD) despite being as or more likely than high-income children to meet the diagnostic criteria for this condition [45]. Because all children had access to publicly funded health care and prescription drugs during our study period, it is possible that differential access to supportive specialty services contributes to the observed socioeconomic disparities in treatment. Although speculative, this assertion is supported by the lack of association between the density of publicly funded child and adolescent psychiatrists and developmental pediatricians with psychotropic dispensing rates, but higher rates of stimulant use and lower rates of antipsychotic use with an increasing density of mental health facilities providing comprehensive diagnostic assessments and access to allied health professionals and non-pharmacologic therapies that are not otherwise covered if not accessed through these organizations. Notably, treatment guidelines for most disorders in children and youth, with the exception of disorders producing psychosis, recommend starting with non-pharmacologic behavioural or psychological therapies [46–48]. However, wait times for publicly-funded child and youth mental health services in Ontario have increased considerably, potentially delaying access to non-pharmacologic therapy and diagnostic assessment [49]. This may exacerbate income-related disparities in psychotropic medication use because higher-income families have the means to access non-pharmacologic mental health care through private funding, forcing low-income families to rely on medical settings for care in which the mainstay of treatment is medication. Prior research demonstrating that fewer than half of children and youth dispensed antipsychotics received non-pharmacological services in the preceding 90 days supports the notion of pharmacologic substitution with antipsychotics for managing non-psychotic conditions in children and youth [11].

Implicit bias on the part of healthcare professionals, educators and child-care workers, in which the behaviours of low-income children are disproportionately problematized relative to children from higher-income families, could also contribute to the greater use of antipsychotic drugs in low-income children and youth [50, 51]. This assertion is supported by U.S. research demonstrating a greater likelihood of diagnosis with conduct or oppositional defiance disorders among low-income African-American and Hispanic-American youth relative to non-Hispanic white children and youth, despite exhibiting comparable behaviours [52–56]. However, further research would be required to ascertain whether implicit bias is contributing to the patterns of psychotropic dispensing observed in our study.

Psychotropic dispensing was also influenced by household size and the proportion of the population that were non-Canadian citizens. Specifically, benzodiazepine dispensing rates were higher as the mean number of individuals per private dwelling increased, whereas the converse was true for stimulants. Existing research examining the association between family size and mental health in children and youth is limited and inconclusive, with studies demonstrating protective and deleterious effects of increased family size on the mental health of children and youth [57, 58]. In addition, past research has found a delayed diagnosis of ADHD in households with more children, potentially supporting our findings [59]. Further research examining the association between household size and mental health outcomes is needed. In addition, our finding of an inverse association between stimulant dispensing and the proportion of the population that are not Canadian citizens is similar to prior research demonstrating less use of these drugs among immigrants [60–63]. Whether this reflects less access to diagnostic testing and treatment, implicit bias on the part of health-care providers or teachers, cultural responses to health and illness or a combination of the above requires further study.

Our results have implications for practice and policy. Specifically, our findings associating the nature of psychotropic medication dispensing with the availability of services suggests that increasing access to publicly funded physician-based and non-pharmacologic services is needed to minimize wait times and antipsychotic use. However, cost, staff retention, and the size of the available clinician workforce limit the extent and pace with which system delivery enhancements can be implemented. Clinician education and training programs enabling concordance with treatment guidelines and best practices are alternative approaches for optimizing psychotropic drug use in children and youth, with several programs in the United States demonstrating improved prescribing appropriateness for stimulants and antipsychotics [64, 65]. A similar approach has been implemented in Ontario through Project Extension of Community Health Outcomes (ECHO) [66]. Project ECHO Ontario uses an innovative continuing professional education model to train health care providers throughout the province to provide specialist-level care to children and youth with mental health and neurodevelopmental conditions, with over 700 providers being trained since 2016. Evaluation of the program is ongoing.

Some limitations of our work merit emphasis. First, we did not have access to various individual-level characteristics that have been previously identified as determinants of psychotropic use, including contact with the child welfare system, race, and juvenile justice system involvement. Similarly, we lacked information regarding regional variation in illness, school-based interventions and the regional availability of community-based social workers, psychologists, and other professionals in private practice specializing in the care of children and youth with mental health and neurodevelopmental conditions. Our analyses should therefore be considered descriptive, with further research needed that considers these additional variables to better understand the observed variations. Second, we cannot ascertain whether variation in psychotropic rates represents overuse or underuse in specific populations. However, we believe that both may be occurring in the context of stimulant and antipsychotic use given the socioeconomic disparities observed, prior research, and associations with the regional density of mental health services. Finally, our study was conducted in a single Canadian province in the year universal funding of medications for all children and youth was introduced, potentially limiting the generalizability of our findings. However, our study includes all children and youth dispensed psychotropic drugs in a setting of publicly funded healthcare. Our findings of disparities that persist despite universal health care may be transferable to similar settings where prescription drugs are provided at no cost to children and youth.

## Conclusions

In summary, we found considerable variation and potential disparities in psychotropic use among children and youth across Ontario. Most notably, socioeconomic gradients in antipsychotic use highlight the possibility of systemic inequity in access to non-pharmacological behavioural and psychosocial interventions despite universal pharmacare and publicly funded access to physician specialists. Targeted investment in regions with long wait times for publicly funded non-pharmacological interventions and novel service delivery models promoting clinician collaboration and education may help minimize disparities and promote best practices in psychotropic drug prescribing to children and youth.

## Supplementary Information

**Additional file 1: Supplemental Fig. S1.** Age and sex adjusted rates of individuals dispensed antidepressants by census division (per 1000 population). **Supplemental Fig. S2.** Age and sex adjusted rates of individuals dispensed stimulants by census division (per 1000 population). **Supplemental Fig. S3.** Age and sex adjusted rates of individuals dispensed benzodiazepines by census division (per 1000 population). **Supplemental Fig. S4.** Age and sex adjusted rates of individuals dispensed antipsychotics by census division (per 1000 population).

**Additional file 2: Supplemental Table 1.** Baseline Variables by Drug Class. **Supplemental Table 2.** Small area variation analysis of psychotropic prescribing by drug class. **Supplemental Table 3.** Diagnosis of schizophrenia and other antipsychotic disorders or autism spectrum disorder in the 30 and 365 days preceding antipsychotic dispensing.

## Data Availability

The data set from this study is not available publicly and is held securely in coded form at ICES. While data sharing agreements prohibit ICES from making the data set publicly available, access may be granted to those who meet pre-specified criteria for confidential access, available at www.ices.on.ca/DAS. The full data set creation plan and underlying analytic code are available from the authors upon request, understanding that the programs may rely upon coding templates or macros that are unique to ICES.

## Abbreviations

EQ: Extremal quotient
CV: Coefficient of variation
SCV: Systematic component of variation
ADGs: Aggregated diagnosis groups
RR: Rate ratio
CIs: Confidence intervals
IQR: Interquartile range
SD: Standardized difference
ADHD: Attention-deficit hyperactivity disorder
ECHO: Extension of Community Health Outcomes
