# Supplementary material for: Geographic variation and sociodemographic correlates of prescription psychotropic drug use among children and youth in Ontario, Canada: a population-based study
Source: BMC Public Health. 2023 Jan 11;23:85. doi: 10.1186/s12889-022-14677-6 (PMC9832754; doi:10.1186/s12889-022-14677-6)

**Figure Legend**

Supplemental Figure S1: Age and sex adjusted rates of individuals dispensed antidepressants by census division (per 1,000 population)

Supplemental Figure S2: Age and sex adjusted rates of individuals dispensed stimulants by census division (per 1,000 population)

Supplemental Figure S3: Age and sex adjusted rates of individuals dispensed benzodiazepines by census division (per 1,000 population)

Supplemental Figure S4: Age and sex adjusted rates of individuals dispensed antipsychotics by census division (per 1,000 population)

Supplemental Figure S1: Age and sex adjusted rates of individuals dispensed antidepressants by census division (per 1,000 population)


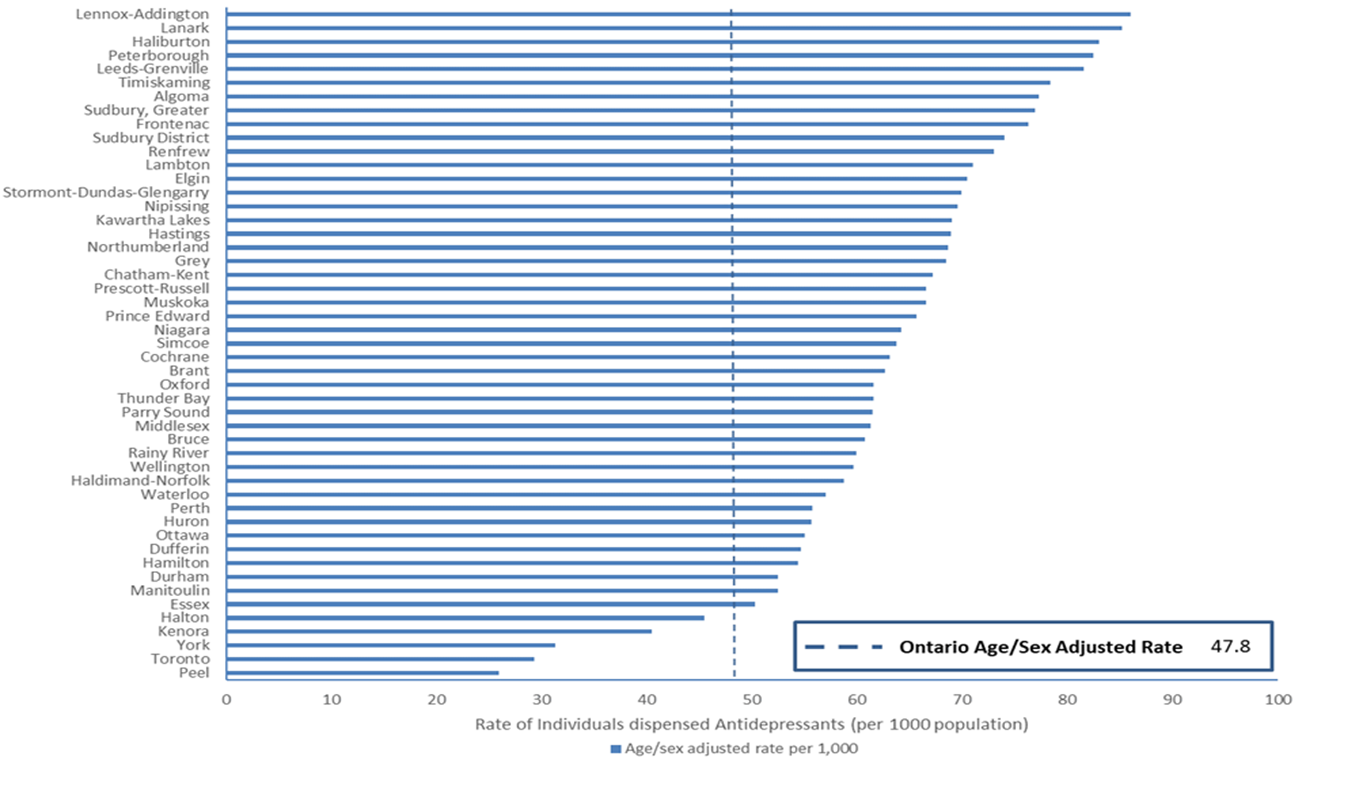


Supplemental Figure S2: Age and sex adjusted rates of individuals dispensed stimulants by census division (per 1,000 population)


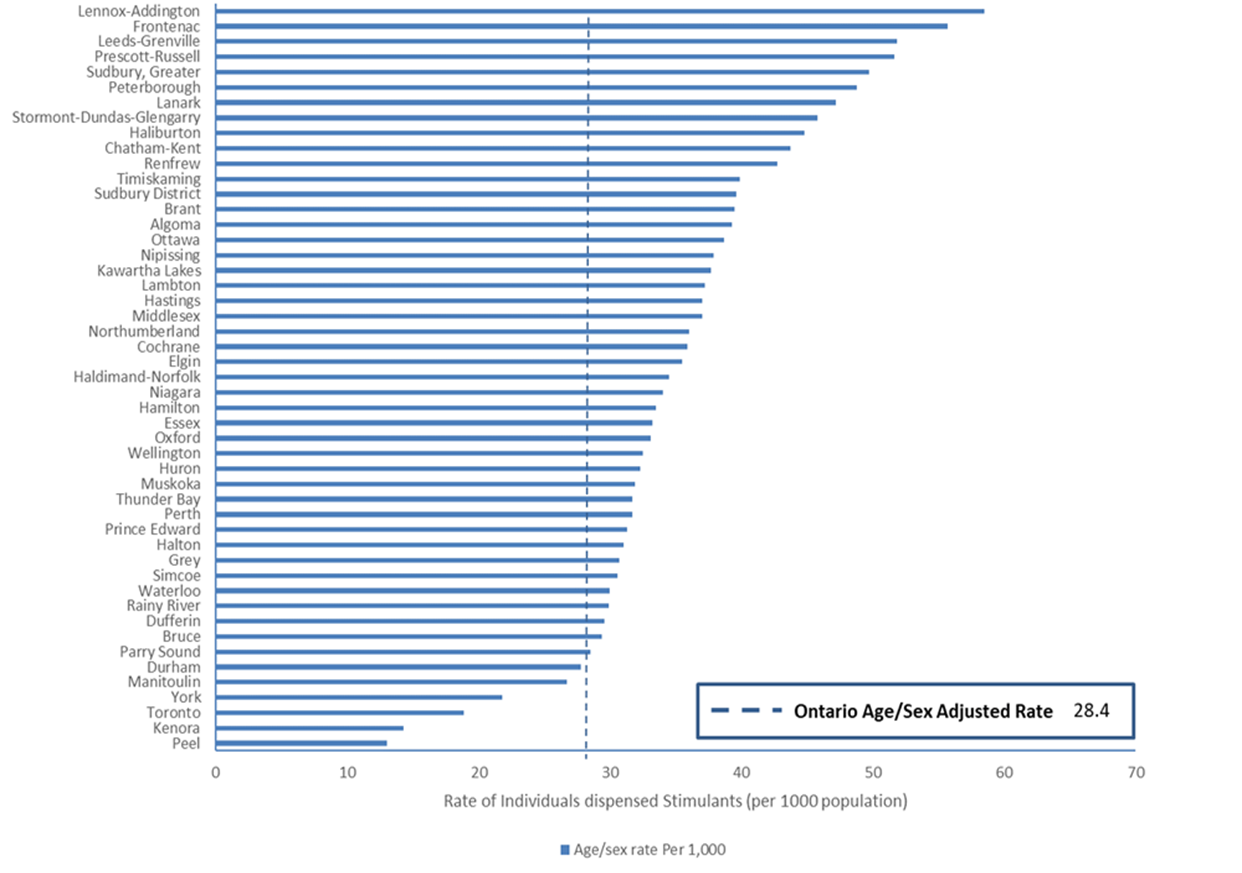


Supplemental Figure S3: Age and sex adjusted rates of individuals dispensed benzodiazepines by census division (per 1,000 population)


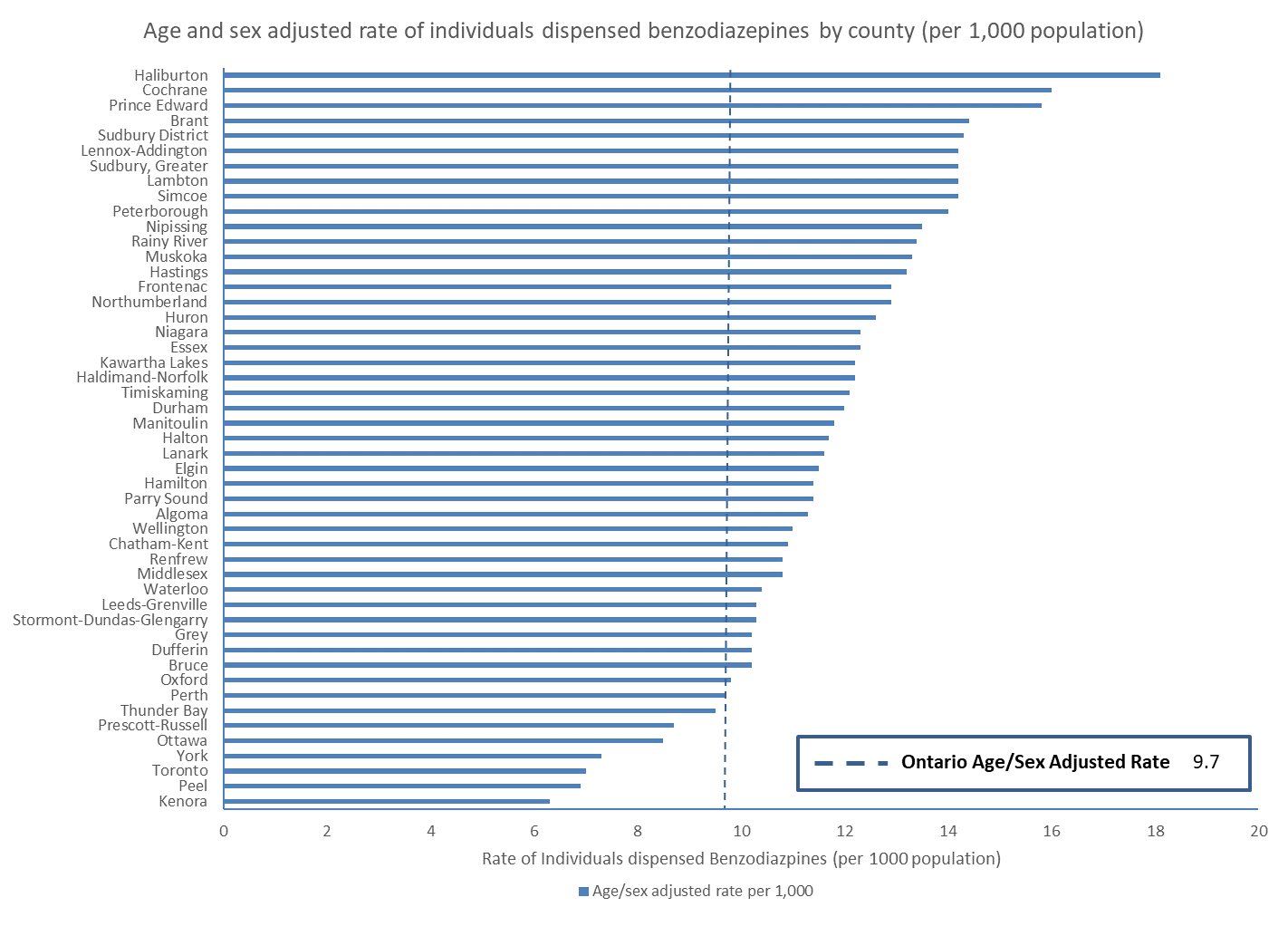


Supplemental Figure S4: Age and sex adjusted rates of individuals dispensed antipsychotics by census division (per 1,000 population)


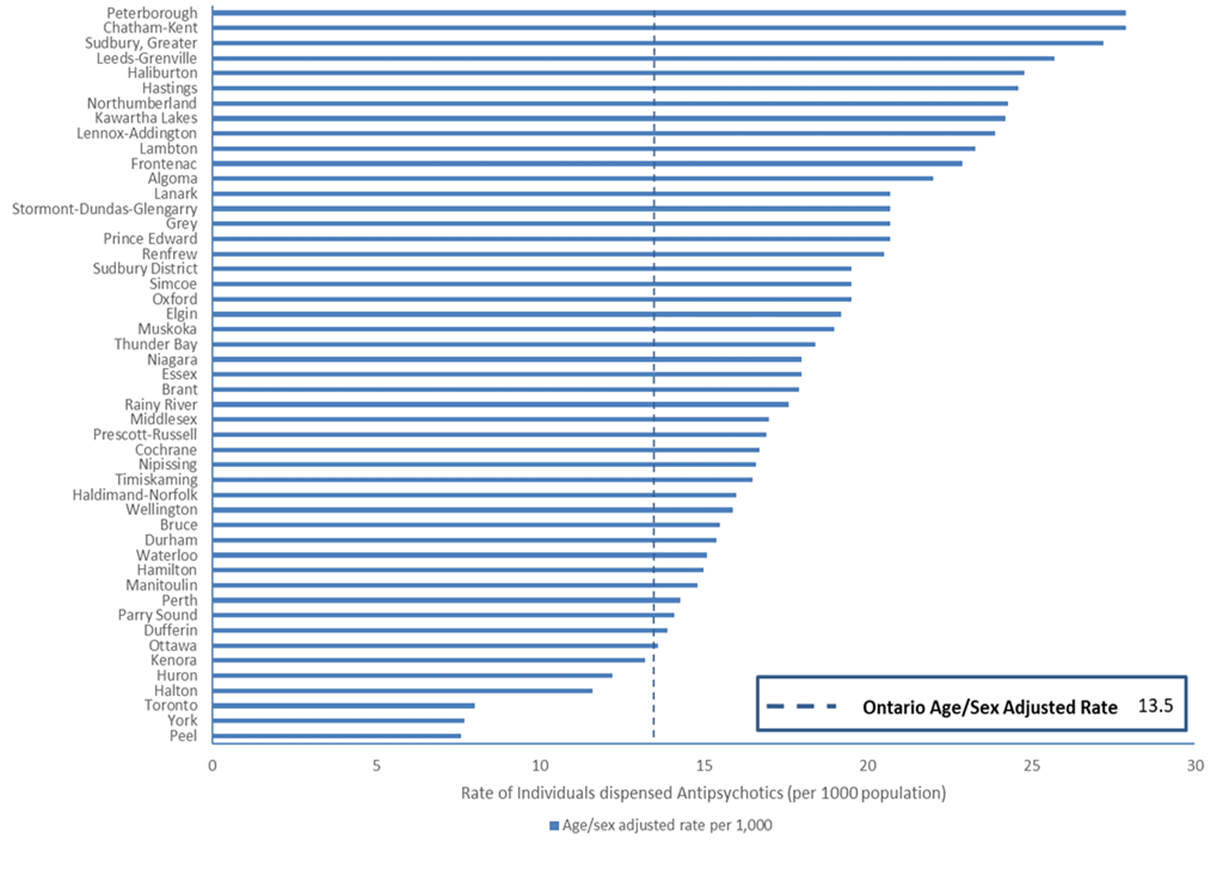

Supplement: Supplementary file 1 — Additional file 1: Supplemental Fig. S1. Age and sex adjusted rates of individuals dispensed antidepressants by census division (per 1000 population). Supplemental Fig. S2. Age and sex adjusted rates of individuals dispensed stimulants by census division (per 1000 population). Supplemental Fig. S3. Age and sex adjusted rates of individuals dispensed benzodiazepines by census division (per 1000 population). Supplemental Fig. S4. Age and sex adjusted rates of individuals dispensed antipsychotics by census division (per 1000 population). [file 12889_2022_14677_MOESM1_ESM.docx]
