# Supplementary material for: Geographic variation and sociodemographic correlates of prescription psychotropic drug use among children and youth in Ontario, Canada: a population-based study
Source: BMC Public Health. 2023 Jan 11;23:85. doi: 10.1186/s12889-022-14677-6 (PMC9832754; doi:10.1186/s12889-022-14677-6)
Supplement: Supplementary file 2 — Additional file 2: Supplemental Table 1. Baseline Variables by Drug Class. Supplemental Table 2. Small area variation analysis of psychotropic prescribing by drug class. Supplemental Table 3. Diagnosis of schizophrenia and other antipsychotic disorders or autism spectrum disorder in the 30 and 365 days preceding antipsychotic dispensing. [file 12889_2022_14677_MOESM2_ESM.docx]

Supplemental Table 1: Baseline Variables by Drug Class

| **Variable** | **All Psychotropics**  **(n = 306,470)** | **Antidepressants**  **(n = 187,313)** | **Antipsychotics**  **(n = 53,782)** | **Benzodiazepines**  **(n = 37,714)** | **Stimulants**  **(n = 117,167)** |
| --- | --- | --- | --- | --- | --- |
| Age (median, IQR) | 18 (14 to 21) | 20 (17 to 22) | 19 (15 to 22) | 21 (18 to 23) | 14 (10 to 18) |
| 0-4 | 1,175 (0.4%) | 69 (0.0%) | 326 (0.6%) | 200 (0.5%) | 723 (0.6%) |
| 5-9 | 30,060 (9.8%) | 2,847 (1.5%) | 4,671 (8.7%) | 558 (1.5%) | 27,087 (23.1%) |
| 10-14 | 50,826 (16.6%) | 16,751 (8.9%) | 7,985 (14.8%) | 1,884 (5.0%) | 36,355 (31.0%) |
| 15-19 | 98,517 (32.1%) | 70,930 (37.9%) | 16,815 (31.3%) | 11,481 (30.4%) | 29,626 (25.3%) |
| 20-24 | 125,892 (41.1%) | 96,716 (51.6%) | 23,985 (44.6%) | 23,591 (62.6%) | 23,376 (20.0%) |
| Female, No. (%) | 159,740 (52.1%) | 121,544 (64.9%) | 24,909 (46.3%) | 23,830 (63.2%) | 36,567 (31.2%) |
| Income quintile |  |  |  |  |  |
| 1 (lowest) | 62,279 (20.3%) | 38,543 (20.6%) | 13,963 (26.0%) | 8,039 (21.3%) | 22,121 (18.9%) |
| 2 | 57,947 (18.9%) | 36,225 (19.3%) | 11,094 (20.6%) | 7,307 (19.4%) | 21,006 (17.9%) |
| 3 | 57,269 (18.7%) | 35,180 (18.8%) | 9,909 (18.4%) | 7,118 (18.9%) | 21,542 (18.4%) |
| 4 | 60,796 (19.8%) | 36,831 (19.7%) | 9,487 (17.6%) | 7,283 (19.3%) | 23,955 (20.4%) |
| 5 | 68,179 (22.2%) | 40,534 (21.6%) | 9,329 (17.3%) | 7,967 (21.1%) | 28,543 (24.4%) |
| Residence |  |  |  |  |  |
| Urban | 271,467 (88.6%) | 165,534 (88.4%) | 47,683 (88.7%) | 33,866 (89.8%) | 104,374 (89.1%) |
| Rural | 35,003 (11.4%) | 21,779 (11.6%) | 6,099 (11.3%) | 3,848 (10.2%) | 12,793 (10.9%) |
| ADG Category |  |  |  |  |  |
| 0-5 | 147,812 (48.2%) | 82,190 (43.9%) | 20,074 (37.3%) | 13,302 (35.3%) | 64,409 (55.0%) |
| 6-9 | 111,880 (36.5%) | 71,055 (37.9%) | 20,848 (38.8%) | 14,583 (38.7%) | 40,628 (34.7%) |
| > 10 | 46,778 (15.3%) | 34,068 (18.2%) | 12,860 (23.9%) | 9,829 (26.1%) | 12,130 (10.4%) |
| Other psychotropic |  | 61,267 (32.7%) | 41,378 (76.9%) | 26,450 (70.1%) | 33,489 (28.6%) |

Supplemental Table 2: Small area variation analysis of psychotropic prescribing by drug class

| **Drug class** | **Extremal quotient** | **Coefficient of Variation** | **Systematic component of variation** |
| --- | --- | --- | --- |
| Antidepressants | 3.32 | 35.31 | 183.79 |
| Antipsychotics | 3.68 | 40.69 | 261.34 |
| Benzodiazepines | 2.87 | 26.54 | 96.26 |
| Stimulants | 4.49 | 35.85 | 184.25 |

Supplemental Table 3: Diagnosis of schizophrenia and other antipsychotic disorders or autism spectrum disorder in the 30 and 365 days preceding antipsychotic dispensing

|  | **Number children and youth dispensed an antipsychotic** | **Schizophrenia and other psychotic disorders (30 days prior to antipsychotic dispensing)** | **Schizophrenia and other psychotic disorders (365 days prior to antipsychotic dispensing)** | **Autism spectrum disorder (30 days prior to antipsychotic dispensing)** | **Autism spectrum disorder (365 days prior to antipsychotic dispensing)** |
| --- | --- | --- | --- | --- | --- |
| Income quintile 1 (lowest) | 13,963 | 2,414 (17.3%) | 4,851 (34.7%) | 421 (3.0%) | 1,241 (8.9%) |
| Income quintile 2 | 11,094 | 1,824 (16.4%) | 3,748 (33.8%) | 352 (3.2%) | 1,044 (9.4%) |
| Income quintile 3 | 9,909 | 1,686 (17.0%) | 3,499 (35.3%) | 333 (3.4%) | 986 (10.0%) |
| Income quintile 4 | 9,487 | 1,492 (15.7%) | 3,148 (33.2%) | 318 (3.4%) | 970 (10.2%) |
| Income quintile 5 | 9,329 | 1,503 (16.1%) | 3,141 (33.7%) | 307 (3.3%) | 964 (10.3%) |
